# The therapeutic effect of acupuncture in the treatment of chemotherapy-induced peripheral neuropathy: a randomized controlled trial

**DOI:** 10.3389/fonc.2025.1500410

**Published:** 2025-04-16

**Authors:** Jinqun Xu, Qing Zhang, Na Xue, Cui Zhang, Weiru Xu

**Affiliations:** ^1^ Department of Oncology, Beijing Hospital of Traditional Chinese Medicine, Capital Medical University, Beijing, China; ^2^ Department of Oncology, Yibin Hospital of Traditional Chinese Medicine, Yibin, China

**Keywords:** acupuncture, chemotherapy, peripheral neuropathy, therapeutic, randomized controlled trial

## Abstract

**Introduction:**

Effective treatments for preventing and treating chemotherapy-induced peripheral neuropathy (CIPN) are still under exploration. Acupuncture in the treatment of CIPN requires more clinical trial data. This study aimed to evaluate the therapeutic effect of acupuncture on CIPN and explore its efficacy and safety in improving peripheral neuropathy.

**Methods:**

A randomized controlled trial was conducted from May 2021 to June 2023. Eligible patients were randomly divided into a verum acupuncture group and a sham acupuncture group at a 1:1 ratio with sealed opaque envelopes. The patients of both the two groups took oral mecobalamin tablets, 0.5 mg, three times a day for 2 weeks. Participants received acupuncture treatment three times a week for 2 weeks. The primary outcome was evaluated using the National Cancer Institute Common Terminology Criteria for Adverse Events (NCI-CTCAE) 3.0. The secondary outcomes were assessed using the European Organization for Research and Treatment of Cancer (EORTC) Quality of Life Questionnaire-CIPN twenty-item subscale (QLQ-CIPN20), Numerical Rating Scale (NRS), Traditional Chinese Medicine (TCM) syndrome score, and nerve conduction study (NCS) testing. Assessments were conducted at baseline, 1 week, and 2 weeks.

**Results:**

All 70 participants were recruited and randomized. In the end, 68 patients were included in the datasets and received verum acupuncture (n = 34) or sham acupuncture (n = 34). After 2 weeks of treatment and follow-up, a statistically significant difference was found in the NCI-CTCAE scores between the two groups (p = 0.02). Baseline-to-2-week assessment scores improved significantly in the intervention group (vs. controls) on EORTC QLQ-CIPN20 (p = 0.02), NRS scores (p = 0.03), TCM syndrome scores (p = 0.04), and sensory nerve action potential (SNAP) of median and peroneal nerves, sensory nerve conduction velocity (SNCV) and motor nerve conduction velocity (MNCV) of peroneal nerves (p < 0.05). No serious adverse events were reported.

**Conclusion:**

This study supports the feasibility of acupuncture combined with medication as an intervention for patients with CIPN and confirms its efficacy and safety in improving peripheral neuropathy.

**Clinical Trial Registration:**

The Chinese Clinical Trial Registry, identifier ChiCTR2100045762.

## 1 Introduction

Chemotherapy-induced peripheral neuropathy is a serious adverse effect and occurs after the usage of chemotherapeutic agents (1). Damage of peripheral neuropathy usually causes sensory and motor deficits, characterized by hyperpathia, tingling, numbness, and weakness in the hands and/or feet (2–4). The prevalence of CIPN was more than 68% (5–7). Platinum-based drugs have the highest incidence of peripheral neuropathy (70%–100%), and taxane-induced peripheral neuropathy ranks second (11%–87%) (8). Symptoms are dose dependent and would lead to discontinuation of treatment or reduction of treatment dose affecting the patient’s overall survival (9). Some patients could pursue CIPN for months after treatment discontinuation, which severely influences their quality of life physically and financially (10, 11). Therefore, it is extremely necessary to actively and effectively treat CIPN as early as possible to improve the quality of life and effectiveness of treatment for cancer patients.

Currently, treatment of CIPN includes nerve-protective therapy, ion channel-targeted therapy, anti-inflammatory therapy, neurotransmitter-based therapy, antioxidant, and non-drug treatment (12), whereas effective treatments for preventing and treating CIPN are still under exploration because of the varying pathophysiology of CIPN with different classes of chemotherapy drugs (5, 13),. According to the latest American Society of Clinical Oncology (ASCO) guidelines, duloxetine is the only drug with evidence to support its use in the treatment of painful CIPN (14). Duloxetine belongs to a class of medications called selective serotonin and norepinephrine reuptake inhibitors (SNRIs), it is likely to enhance analgesia but is not neuroprotective (15). Current clinical treatment recommendations for CIPN include analgesia and symptom management. Some studies have shown that acupuncture can effectively reduce chemotherapy-induced peripheral neuropathy in patients with hyperpathia and other various symptoms (16, 17). However, Traditional Chinese Medicine (TCM) syndromes have not yet been involved in current studies on acupuncture treatment of CIPN. Syndrome differentiation, as a fundamental approach in TCM diagnosis and treatment, can guide the selection of acupuncture points (18, 19). In this study, we aimed to explore the efficacy and safety of acupuncture combined with medication in the treatment of CIPN with the syndrome of qi deficiency and blood stasis and provide an alternative direction for the prevention and treatment of CIPN in the future. We believe that the effective rate of acupuncture for CIPN can be increased under the guidance of TCM evidence-based treatment. It will also help doctors to accurately treat the patients with the syndrome of qi deficiency and blood stasis. Besides, in this study, we used multi-scales to comprehensively analyze the effectiveness of acupuncture in treating CIPN, including patients’ subjective symptoms (NCI-CTCAE, EORTC QLQ-CIPN20, NRS), objective data (NCS), and the syndrome score of Traditional Chinese Medicine.

## 2 Methods

### 2.1 Study design

We conducted a single-center, randomized, controlled, single-blind clinical trial. The therapeutic verum acupuncture group was compared to the sham acupuncture group for patients with chemotherapy-induced peripheral neuropathy (CIPN). Patients were randomly divided into a verum acupuncture group and a sham acupuncture group. We observed both groups to evaluate the therapeutic effect of acupuncture combined with medication on CIPN and explored its efficacy and safety in improving peripheral neuropathy in patients. This study aimed to establish a treatment method that can be clinically applied and promoted.

### 2.2 Recruitment and inclusion criteria

We conducted the study at the Beijing Hospital of Traditional Chinese Medicine, Capital Medical University. Participants were recruited at the outpatient clinic and inpatient ward of the Oncology Department. The study coordinator explained the possible benefits and adverse reactions of the trial to the eligible patients, and all patients signed informed consent forms.

Inclusion criteria included the following:

1. All cases were confirmed as malignant tumors by histopathology or cytology.
2. Patients underwent chemotherapy with platinum-based or taxane agents and subsequently developed sensor neuropathy (numbness, sensory loss, pain, paresthesia in the extremities) and/or motor neuropathy (muscle cramps, muscle weakness, myalgia, paralysis).
3. Patients rated grade 2 or higher on the National Cancer Institute Common Terminology Criteria for Adverse Events (NCI-CTCAE) for sensory and motor neuropathy within 7 days to 2 years after cessation of chemotherapy.
4. Patients were diagnosed with qi deficiency and blood stasis as the primary syndrome according to Traditional Chinese Medicine (TCM) differentiation.
5. Patients with Eastern Cooperative Oncology Group (ECOG) performance status scores of 0–3.
6. Patients with an expected survival time of more than 3 months.

Exclusion criteria included the following:

1. Patients with peripheral neuropathy that were not caused by chemotherapy agents.
2. Patients were undergoing treatment with other medications that may cause neurotoxicity.
3. Patients who received any suspected effective medication, such as tricyclic antidepressants or anticonvulsants.
4. Patients with severe diseases involving the cardiovascular, hepatic, renal, immune, or hematopoietic systems.
5. Patients were pregnant or breastfeeding.
6. Patients cannot undergo acupuncture treatment, due to needle phobia or allergy to stainless steel needles, etc.

### 2.3 Randomization and blinding

Eligible patients were randomly allocated to the verum acupuncture group or sham acupuncture group in a 1:1 ratio. An independent researcher at the Beijing Hospital of Traditional Chinese Medicine generated random numbers by a computer-generated randomization program. Random sequences were loaded into sealed opaque envelopes and kept by the study coordinator. All neuropathy evaluations were performed exclusively by a trained medical professional, who had underwent standardized training to ensure consistency and adherence to the NCI-CTCAE guidelines prior to patient enrollment. The acupuncturist did not participate in the patient’s inclusion. The study coordinator opened the envelope and informed the acupuncturist before the intervention. The study participants, assessor, and statistician were all blinded to the treatment assignments. Only the acupuncturist and study coordinator were aware of these details.

### 2.4 Intervention

All acupuncture procedures were performed by the same licensed acupuncturist with over 5 years of experience. During the treatment, patients were asked to avoid medications that might alleviate CIPN (such as tricyclic antidepressants or antiepileptic drugs, Chinese herbal decoction, and patent Chinese medicine).

**Figure 1:**
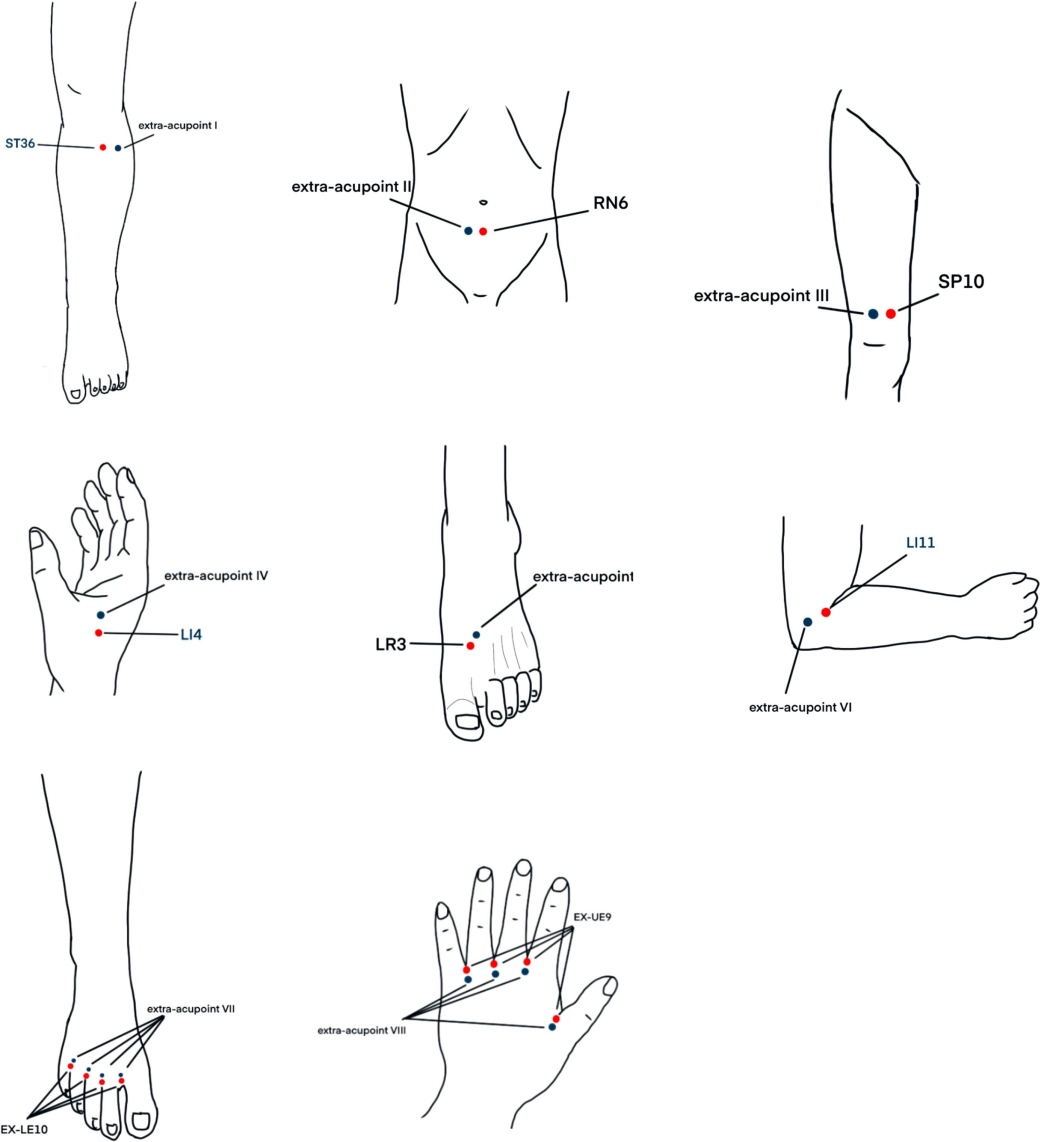
The acupoints of the verum acupuncture group and the sham acupuncture group.

Participants in the verum acupuncture (intervention) group received 2 weeks of acupuncture treatment. The principle of acupuncture treatment was activating meridians and collaterals, benefiting qi, and promoting blood circulation to remove blood stasis. The acupuncture prescription included bilateral ST36, SP10, LI4, LR3, LI11, EX-LE10, EX-UE9, and RN6. The location of the acupoints are illustrated in **Figure 1**. The acupuncturist followed the guidelines outlined in the *Acupuncture and Moxibustion Medicine* from the New Century Higher Medical Education Planning Textbook series (published by China Traditional Chinese Medicine Press). The acupuncturist used disposable sterile acupuncture needles (0.25 × 40 mm, 0.25 × 25 mm, Hwato, China), then disinfected the acupuncture points and their hands strictly with 75% alcohol before needle insertion.

After needle insertion, each acupoint was evenly lifted, thrust, and twirled three times to achieve a local sensation of soreness, numbness, distension, or heaviness indicating “*de qi*.” The needles were retained for 30 min, with one needle twisted and performed midway. Treatment frequency is three times per week, lasting for 2 weeks.

Participants in the sham acupuncture (control) group received 2 weeks of sham acupuncture treatment. This group used non-traditional acupoints located adjacent to the conventional acupoints. Extra-acupoint I was outwardly parallelled 1 cun with ST36, extra-acupoint II was outwardly parallelled 1 cun with RN6, extra-acupoint III was outwardly parallelled 1 cun with SP10, extra-acupoint IV was the midpoint between LI4 and EX-UE9, extra-acupoint V was the midpoint between LR3 and ST42, extra-acupoint V was the midpoint between TE10 and LI11, extra-acupoint VII was upward 0.25 cun from EX-LE10, and extra-acupoint VII was upward 0.25 cun from EX-UE9. The location of the acupoints are illustrated in **Figure 1**. The needles were inserted superficially and penetrated only the subcutaneous layer without manipulation to obtain *qi*. The acupuncture instruments, needle insertion frequency, and treatment duration were consistent with those of the verum acupuncture group.

The patients of both the two groups received medication treatment: oral mecobalamin tablets (Produced by Eisai China Pharmaceutical Co., Ltd., approval number: National Medicine Standard H20143107), 0.5 mg, three times a day, continuously for 2 weeks.

### 2.5 Outcome measures

The patient’s general condition and tumor-related indicators were recorded before the time of random assignment, including age, sex, body mass index (BMI), employment status, education, diagnosis, cancer stage, adjuvant type, chemotherapy cycles, ECOG performance status, months after last chemotherapy. Outcomes related to preliminary efficacy included primary outcome indicators and secondary outcome indicators. The independent assessor recorded relevant outcomes at baseline, 1 week, and 2 weeks after initiation of the study intervention.

#### 2.5.1 Primary outcomes

Peripheral neuropathy grading based on NCI-CTCAE scale V3.0 was used to evaluate the primary outcome.

#### 2.5.2 Secondary outcomes

Secondary outcomes were evaluated using the European Organization for Research and Treatment of Cancer (EORTC) Quality of Life Questionnaire-CIPN twenty-item subscale (QLQ-CIPN20), Numerical Rating Scale (NRS), Traditional Chinese Medicine(TCM) syndrome score, and nerve conduction study (NCS) testing.

The EORTC QLQ-CIPN20 evaluates the sensory, motor, and autonomic symptoms of peripheral nerves (20). The assessor scored according to the actual situation, with a four-point system for each item. The higher the total score, the worse the peripheral nerve function. The NRS was used to assess pain severity using numbers from 0 to 10, where 0 represents no pain and 10 represents the worst pain imaginable.

The TCM syndrome is qi deficiency and blood stasis syndrome. The respondents scored according to the actual situation, with a four-point method for each item. The higher the total score, the worse the symptoms related to the TCM syndrome. The diagnosis of this syndrome was based on *the Guidelines for Clinical Research of New Traditional Chinese Medicine Drugs for Syndrome Patterns issued by the National Medical Products Administration* (21). According to this guideline, a patient is diagnosed with “qi deficiency and blood stasis” syndrome if they meet one of the following criteria: 1. One primary symptom of qi deficiency plus one primary symptom of blood stasis. 2. One primary symptom of qi deficiency plus one secondary symptom of blood stasis. 3. One primary symptom of blood stasis plus one secondary symptom of qi deficiency. Additionally, the diagnosis requires specific tongue and pulse signs.

Primary symptoms: 1. Qi deficiency: shortness of breath, weakness, fatigue. 2. Blood stasis: stabbing pain, fixed pain, tenderness, vascular stasis, subcutaneous bruising, abdominal masses, or bleeding.

Secondary symptoms: 1. Qi deficiency: spontaneous sweating, lazy speech. 2. dry or scaly skin, limb numbness or paralysis, mental disorders, irritability, forgetfulness, localized sensory abnormalities, or a history of trauma, surgery, or abortion.

Tongue and pulse: purple and dark tongue or petechiae or ecchymosis, sublingual varices, or pale tongue; unsmooth pulse, no pulse, sinking and string pulse, string and slow pulse, or deficient pulse.

Patients underwent nerve conduction study (NCS) testing by independently trained neurologists before and after treatment. Skin surface electrodes were tested with Viking Quest, Nicolet EDX, EMG/NCS/EP/IOM System (Natus, America). We studied the motor conduction of the bilateral median and peroneal nerves by recording the motor conduction velocity and the amplitude. Sensory conduction was tested in the bilateral median and peroneal nerves by measuring the sensory conduction velocity and amplitude. The endpoint was the change in sensory nerve action potential (SNAP), sensory nerve conduction velocity (SNCV), motor nerve action potential (MNAP), and motor nerve conduction velocity (MNCV) in the median and peroneal nerves.

#### 2.5.3 Safety assessment

The study coordinator monitored treatment-related adverse events via standard adverse event (CTCAE v.5.0) reporting throughout the study. Included: 1. Patients underwent blood routine, urine routine, stool routine, liver and kidney functions, and electrocardiogram before and after treatment. 2. Patients were evaluated for acupuncture treatment safety (needle fainting, infection, bleeding, hematoma, needle breakage, and other injuries).

### 2.6 Statistical analysis

EpiData was used to establish the database. Two data managers were responsible for data entry and verification. The statistician performed data statistical analysis.

Statistical analysis was performed using the SAS 9.4 software. If the continuous outcomes obey the normal distribution, we described it as the mean values ± standard deviation. Otherwise, it was expressed as the median, interquartile range, and range. For comparison between the two groups, we used the independent samples t-test. For comparison before and after treatment, we used the paired t-test or the Wilcoxon signed-rank test. Categorical data are expressed as frequencies and percentages. We used the Pearson chi-square test or Fisher’s exact test. All statistical tests used two-sided tests unless otherwise stated, and the significance level was p-value <0.05.

## 3 Result

### 3.1 Datasets and general information

**Figure 2:**
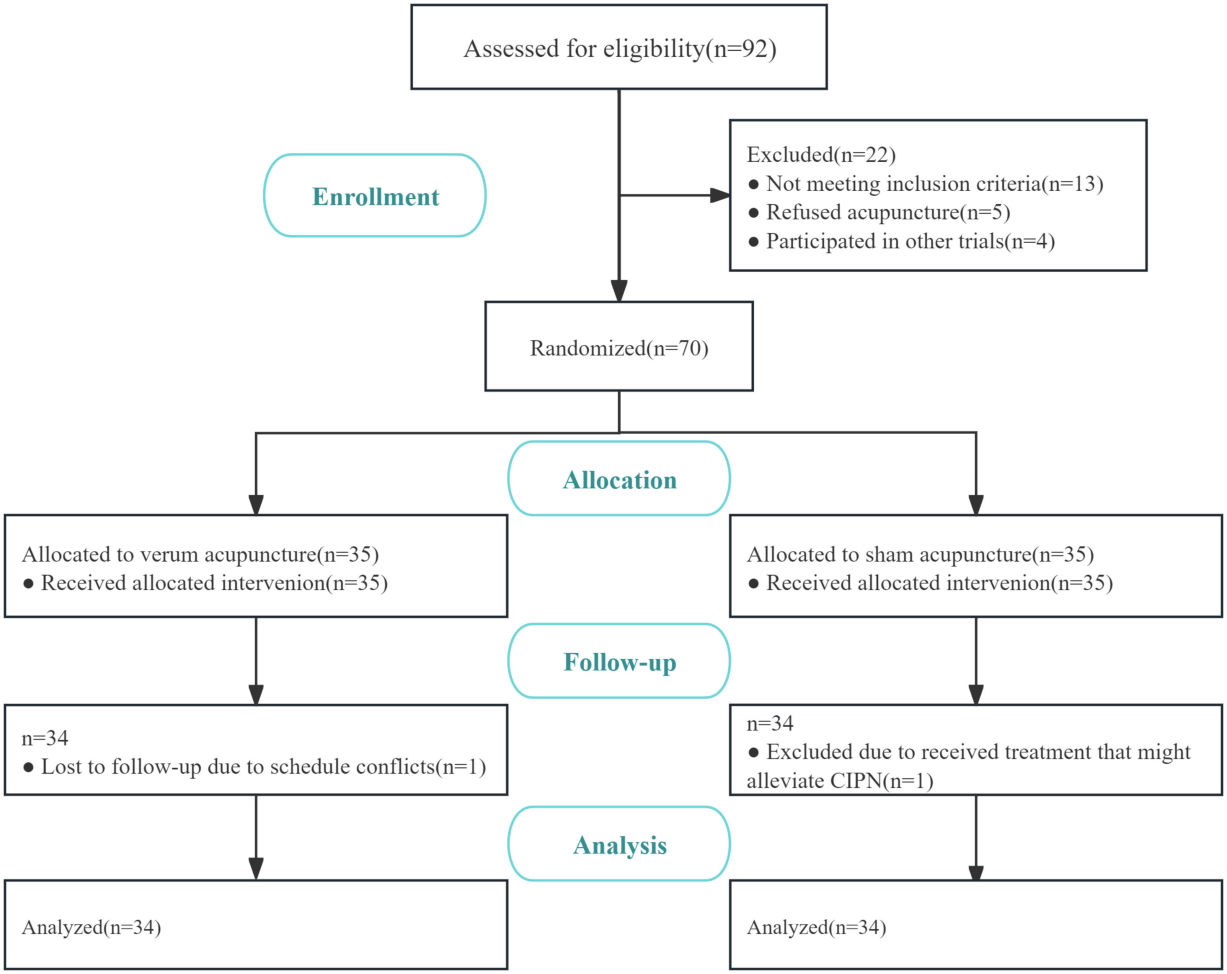
Flow diagram of the study participants.

All 70 participants were recruited at the Beijing Hospital of Traditional Chinese Medicine, Capital Medical University, from January 2021 to June 2023. Thirty-five patients were assigned to the verum acupuncture group, and 35 patients were assigned to the sham acupuncture group. In the verum acupuncture group, one patient was lost to follow-up because of schedule conflicts. In the sham acupuncture group, one patient was excluded because of depression and received treatment that might alleviate CIPN. In our study, patients who missed two follow-up assessments were dropped and excluded from the final analysis. To ensure that patients took methylcobalamin on time and in the correct dosage, as well as to prevent unauthorized use of other medications, we implemented the following measures: 1. Patient and family education. 2. Medication record cards. 3. Monitoring and verification. Finally, 68 patients were included in the datasets. The study process is illustrated in **Figure 2**. We found no statistical differences between the intervention group and control group in the baseline characteristics or clinical characteristics, such as age, sex, BMI, employment status, education, diagnosis, cancer stage, adjuvant type, chemotherapy cycles, ECOG performance status, and months after last chemotherapy, as shown in **Table 1**.

**Table 1:** Clinical characteristics of participants.

Characteristics	Verum acupuncture group (n = 34)	Sham acupuncture group (n = 34)	All (n = 68)
Age, years; mean ± SD	62.2 ± 8.3	59.1 ± 9.7	60.6 ± 9.1
Sex, n (%)			
Male	10 (29.4)	12 (35.3)	22 (32.4)
Female	24 (70.6)	22 (64.7)	46 (67.6)
BMI; mean ± SD	23.2 ± 2.1	22.3 ± 2.2	22.7 ± 2.1
Employment status, n (%)			
Employment	8 (23.5)	10 (29.4)	18 (26.5)
Retired	26 (76.5)	24 (70.6)	50 (73.5)
Education, n (%)			
Below bachelor degree	25 (73.5)	27 (79.4)	52 (76.5)
Bachelor’s degree or above	9 (26.5)	7 (20.6)	16 (23.5)
Diagnosis, n (%)			
Breast cancer	10 (29.4)	6 (17.6)	16 (23.5)
GI cancer	14 (41.2)	15 (44.1)	29 (42.6)
Lung cancer	3 (8.8)	1 (2.9)	4 (5.9)
Gynecological cancer	6 (17.6)	7 (20.6)	13 (19.1)
Thymic carcinoma	0 (0.0)	1 (2.9)	1 (1.5)
Esophageal cancer	0 (0.0)	2 (5.9)	2 (2.9)
Pancreatic cancer	0 (0.0)	1 (2.9)	1 (1.5)
Peritoneal cancer	1 (2.9)	1 (2.9)	2 (2.9)
Cancer stage, n (%)			
I	2 (5.9)	1 (2.9)	3 (4.4)
II	15 (44.1)	11 (32.3)	26 (38.2)
III	11 (32.3)	14 (41.2)	25 (36.7)
IV	6 (17.6)	8 (23.5)	14 (20.6)
Adjuvant type, n (%)			
Taxanes	13 (38.2)	9 (26.5)	22 (32.4)
Platinum	12 (35.3)	14 (41.2)	26 (38.2)
Taxanes and platinum	9 (26.5)	11 (32.4)	20 (29.4)
Chemotherapy cycles, median (interquartile range)			
Taxanes	8 (5, 8)	5 (4, 8)	7 (4, 8)
Platinum	7 (6, 8)	8 (3, 8)	7.5 (6, 8)
Taxanes and platinum	6 (5.5, 6.5)	4 (4, 8)	6 (4, 6.75)
ECOG PS, n (%)			
0	0 (0.0)	0 (0.0)	0 (0.0)
1	24 (70.6)	23 (67.6)	47 (69.1)
2	9 (26.5)	11 (32.4)	20 (29.4)
3	1 (2.9)	0 (0.0)	1 (1.5)
4	0 (0.0)	0 (0.0)	0 (0.0)
5	0 (0.0)	0 (0.0)	0 (0.0)
Months after the last chemotherapy, median (interquartile range)	4 (1, 10)	3 (1,11)	3 (2, 10)

BMI, body mass index; ECOG PS, Eastern Cooperative Oncology Group performance status.

p > 0.05 for all characteristics.

### 3.2 Primary outcomes

NCI-CTCAE peripheral neuropathy grade was the primary outcome measure of this study. We found no significant difference between the two groups about changes in NCI-CTCAE scores from baseline (p = 0.32), and 1 week (p = 0.53). Significant difference in between-group improvements was in favor of NCI-CTCAE scores from 2 weeks (p = 0.02). Data are summarized in **Table 2**.

**Table 2:** NCI-CTCAE scores.

	Verum acupuncture group (n = 34)	Sham acupuncture group (n = 34)	*χ2*	p-Value
Baseline				
Grade 1	0	0	0.99	0.32
Grade 2	19	23		
Grade 3	15	11		
Grade 4	0	0		
Grade 5	0	0		
1 week				
Grade 1	4	1	2.07	0.53
Grade 2	21	23		
Grade 3	9	10		
Grade 4	0	0		
Grade 5	0	0		
2 weeks				
Grade 1	15	5	7.94	0.02
Grade 2	16	21		
Grade 3	3	8		
Grade 4	0	0		
Grade 5	0	0		

NCI-CTCAE, National Cancer Institute’s Common Terminology Criteria for Adverse Event.

### 3.3 Secondary outcomes

The result, as accessed by the EORTC QLQ-CIPN20, showed no significant differences between the two groups at baseline (p=0.14) and 1 week (p = 0.80), respectively. Significant differences were found between groups for improvements at 2 weeks (p = 0.02). Concurrently, we performed an ANCOVA evaluation, and significant differences were found within the verum acupuncture group (p < 0.001), but not within the sham acupuncture group (p = 0.17), as shown in **Table 3**.

**Table 3:** Symptom scores of secondary outcomes.

Variables	Time	Verum acupuncture group (n = 34)				Sham acupuncture group(n = 34)				p-Value*	*t-*Value	95% CI
		Mean ± SD	*t-*Value	95% CI	p-Value**	Mean ± SD	*t-*Value	95% CI	p-Value**			
EORTC QLQ-CIPN20	Baseline	33.2 ± 7.38	8.13	5.20, 8.67	<0.001	31.14 ± 5.51	1.37	−0.84, 4.61	0.17	0.14	1.32	−1.04, 5.16
	1 week	29.66 ± 6.02				30 ± 4.92				0.80	−0.26	−2.96, 2.27
	2 weeks	23.91 ± 4.29				29.26 ± 5.93				0.02	−0.26	−5.60, −0.39
NRS	Baseline	4.22 ± 2.34	3.62	0.76, 2.66	<0.001	3.89 ± 1.23	1.71	−0.08, 1.05	0.09	0.36	0.76	−0.54, 1.23
	1 week	3.17 ± 1.76				3.80 ± 1.16				0.09	−1.76	−1.33, 0.08
	2 weeks	2.51 ± 1.54				3.26 ± 1.22				0.03	−2.23	−1.40, −0.07
TCM syndrome scores	Baseline	15.43 ± 4.02	4.32	2.07, 5.63	<0.001	14.63 ± 3.50	1.68	−0.23, 2.74	0.12	0.39	0.88	−0.99, 2.59
	2 weeks	11.51 ± 2.53				13.07 ± 3.82				0.04	−2.03	−3.05, −0.02

EORTC QLQ-CIPN20, European Organization for Research and Treatment of Cancer Quality of Life Questionnaire-CIPN twenty-item subscale; NRS, Numerical Rating Scale; TCM, Traditional Chinese Medicine; SD, standard deviation.

*p*-*Values from within-group comparison using mixed between within-subject analysis of variance (ANOVA) or matched sample t-test. **p*-*Values from comparisons between two groups using 653 one-sample t-test.

Symptoms of pain in NRS were not statistically different between groups at baseline (p = 0.36) and 1 week (p = 0.09), respectively. Significant between-group alleviations in favor of pain reduction were found in NRS at 2 weeks (p = 0.03). Significant differences were found within the verum acupuncture group (p < 0.001), but not within the sham acupuncture group (p = 0.09), as shown in **Table 3**.

We found that there were no significant differences between the changes in the two groups in TCM syndrome scores at baseline (p = 0.39). There were significant differences between the improvements of the two groups at 2 weeks (p = 0.04). Significant differences were found within the verum acupuncture group (p < 0.001), but not within the sham acupuncture group (p = 0.12), as shown in **Table 3**.

The result of statistics showed that the nerve conduction velocity (NCV) (except the median motor NCV) in the verum acupuncture group improved significantly (p < 0.05), while improvement in the sham acupuncture group was not observed (p > 0.05). The SNAP of both median and peroneal nerves improved significantly in the intervention group (p < 0.05), while the MNAP did not (p > 0.05). The difference in nerve action potential (NAP) was not statistically significant in the sham acupuncture group (p > 0.05). There were significant differences between the two groups in the SNAP of the median and peroneal nerves after 2 weeks of treatment (p < 0.05). In contrast, there were no significant differences between the two groups in the MNAP of the median and peroneal nerves after treatment (p > 0.05). The changes in peroneal nerve SNCV and MACV showed statistical significance in comparison to those of the control group (p < 0.05), while the changes in the median nerve SNCV and MNCV showed no statistical significance (p > 0.05), as shown in **Table 4**.

**Table 4:** Changes in nerve conduction velocity.

Variables	Time	Verum acupuncture				Sham acupuncture				p*-*Value*	*t-*Value	95% CI
		Mean ± SD	*t-*Value	95% CI	p*-*Value**	Mean ± SD	*t-*Value	95% CI	p*-*Value**			
Median SNAP (μV)	Baseline	3.51 ± 3.34	−2.08	−5.15, −0.06	0.04	4.12 ± 3.58	0.42	−1.73, 2.66	0.67	0.61	−0.52	−2.99, 1.77
	Week 2	6.02 ± 3.86				3.58 ± 2.67				0.04	0.41	−1.71, 2.59
Median SNCV (m/s)	Baseline	49.03 ± 7.26	−2.48	−3.61, −0.27	0.03	48.14 ± 7.38	−0.75	−6.49, 2.97	0.46	0.72	0.36	−4.16, 5.95
	Week 2	50.98 ± 7.25				50.32 ± 6.96				0.79	0.27	−4.24, 5.55
Median MNAP (μV)	Baseline	13.33 ± 2.96	−1.32	−3.91, 0.83	0.19	12.5 ± 2.97	−0.90	−3.49, 1.33	0.37	0.41	0.83	−1.20, 2.87
	Week 2	14.82 ± 3.67				13.57 ± 3.87				0.34	0.97	−1.35, 3.84
Median MNCV(m/s)	Baseline	54.21 ± 5.12	−0.78	−2.98, 1.35	0.44	55.01 ± 5.16	0.52	−2.37, 4.04	0.60	0.64	−0.47	−4.18, 2.59
	Week 2	55.02 ± 4.35				54.18 ± 4.85				0.57	0.56	−2.18, 3.87
Peroneal SNAP (μV)	Baseline	1.7 ± 0.52	−2.42	−0.86, −0.07	0.02	1.87 ± 0.59	0.85	−0.25, 0.61	0.39	0.38	−0.89	−0.55, 0.21
	Week 2	2.16 ± 0.56				1.68 ± 0.64				0.03	2.33	0.06, 0.89
Peroneal SNCV(m/s)	Baseline	51.52 ± 6.24	−2.67	−10.45, −0.64	0.03	48.88 ± 7.01	−0.63	−8.40, 4.56	0.53	0.44	0.79	−4.49, 9.76
	Week 2	57.07 ± 6.56				49.25 ± 7.43				0.04	2.23	0.29, 15.35
Peroneal MNAP (μV)	Baseline	4.33 ± 2.49	−1.16	−3.04, 0.82	0.25	4 ± 2.13	−0.82	−2.29, 0.97	0.42	0.67	0.42	−1.27, 1.93
	Week 2	5.43 ± 2.87				4.65 ± 2.52				0.40	0.85	−1.08, 2.64
Peroneal MNCV(m/s)	Baseline	51.61 ± 6.96	−2.96	−9.77, −1.09	0.02	47.39 ± 4.92	−1.18	−4.27, 1.12	0.24	0.15	1.55	−1.81, 10.24
	Week 2	57.05 ± 8.14				48.92 ± 4.86				0.03	2.63	1.16, 15.09

MNAP, motor nerve action potential; MNCV, motor nerve conduction velocity; SNAP, sensory nerve action potential; SNCV, sensory nerve conduction velocity; SD, standard deviation.

*p*-*Values from within-group comparison using matched sample t-test. **p*-*values from comparisons between two groups using one-sample t-test.

### 3.4 Adverse events

Minor hematomas following needle acupuncture were observed in 2 of 68 patients (2.9%) without requiring medical intervention. All events were classified as mild via standard adverse event.

## 4 Discussion

The findings of this study support the feasibility and safety of acupuncture as an intervention for patients with CIPN. After 2 weeks of treatment and follow-up, statistically significant difference was found in NCI-CTCAE scores between the two groups (p = 0.02). This is further supported by the QLQ-CIPN20 where significant improvements were also seen (23.91 ± 4.29 vs. 29.26 ± 5.93). The QLQ-CIPN20, while having many similar questions as the FACT/GOG-NTX, was used in clinical practice and research to measure CIPN. The 20-item scale reflecting three subscales, including sensory, motor, and autonomic symptoms, and functioning were internally consistent (22, 23). Moreover, the NRS was improved best in the verum acupuncture group before and after treatment. We recruited patients with qi deficiency and blood stasis syndrome (24). The verum acupuncture group showed a significant improvement in the TCM syndrome scores. The NCS could more objectively evaluate the nerve fibers’ repair and structural regeneration (25, 26). The results showed that the NCS (SNAP of median and peroneal nerves, SNCV and MACV of peroneal nerve) testing was improved best in the verum acupuncture group. The results of this study showed that acupuncture treatment effectively reduced the neuropathy grade of CIPN, improved symptoms associated with peripheral neuropathy, relieved pain and improved physical fitness for patients, alleviated the TCM symptoms of patients with qi deficiency and blood stasis syndrome, and increased peripheral nerve conduction study.

We conducted a single-center, randomized, controlled, single-blind clinical trial. This trial was well implemented following its study design in terms of randomization, allocation concealment, and blinding of outcome assessors. Several previous studies lacked sham- or attention-control methods (27–29). The influence of placebo effect could interfere with the results. We tried to minimize the risk of bias by using sham acupuncture in the control group. The acupuncturist was aware of group allocation and was asked to talk less with the patients. We hope that these methods could reduce the potential bias to some extent. Moreover, based on ethical and patient considerations, patients in both the two groups took oral mecobalamin tablets.

Acupuncture treatment of CIPN in animal models has some possible mechanisms, including peripheral and spinal levels, but the available evidence is limited. Studies have shown that the mechanism of acupuncture treatment for CIPN may be related to the activation or inhibition of receptors such as spinal opioid receptors (μ, δ, and κ) (30), α2- and β-adrenoceptors (31), 5-hydroxytryptamine 1A (5-HT1A) receptors (32), cannabinoid receptor, and specific ligand-gated ion channels (33). Zhao et al. (34) showed that electroacupuncture alleviates paclitaxel-neuropathic pain via increased mechanical and thermal sensitivity, and this was accompanied by impaired Nrf2-antioxidant response element (Nrf2-ARE) and upregulation of oxidative signals in the dorsal root ganglion (DRG) of rats. According to current studies, acupuncture can be used to increase peripheral microcirculation and skin blood perfusion (35–37), and may contribute to nerve repair with measurable improvement of the axons or myelin sheaths (38).

There is no record of CIPN in the ancient Chinese medical books. According to its typical symptoms, such as numbness, pain, and muscle weakness, we attribute it to the category of “Bi disease” and “impotence.” According to our preliminary study and references, the syndrome of qi deficiency and blood stasis is the main syndrome of CIPN (39–44). Chemotherapeutic drugs are very toxic products, injuring people’s qi. Qi deficiency is unable to promote the circulation of blood leading to blood stasis and internal obstruction. Then, the skin does not get warmth and moistening from the qi and blood. The appearance of numbness, limb sensory loss, movement disorders and muscle atrophy, and other symptoms, can be attributed to the Chinese medicine range of “Bi disease and impotence.” The ancient Chinese medical text “*Treatment of Bi Disease, Classical Evidence Treatment and Cure*” says: “The stasis of qi and blood for a long time leads to Bi disease,” suggesting that the pathogenesis of Bi disease is stagnation of qi and blood. The Bi disease will eventually turn into impotence after a long time, which means that the qi and blood become seriously stagnated.

The “*Yellow Emperor’s Classic of Internal Medicine*” has the following words: “Treat impotence by taking Yangming meridian,” “Stabbing Yangming meridian produces qi and blood,” and the Yangming meridian is the meridian with a high level of qi and blood, which has the effect of warming the yang and dredging the collaterals, tonifying qi and blood. So acupoints on treating impotence are mostly based on the Yangming meridian. The ST36 point is the lower He-sea point of the stomach meridian of Yangming. According to the “*Yellow Emperor’s Classic of Internal Medicine*”: “If you have got Bi disease or cold for a long time, Sanli point must be taken.” Therefore, Zusanli (ST36) is the important acupoint for the treatment of CIPN combined with Quchi (LI11) and Hegu (LI4), the combined acupoints of hand Yangming meridian, which plays an important role in benefiting qi and warming yang. Taichong (LR3) and Hegu (LI4), the combination of Yangming meridian and Jueyin meridian, one yin and one yang, one zang and one fu, reconcile the yin yang and qi and blood in the upper and lower parts of the body, so that the yin and yang are reconciled, and the meridians are unobstructed. The RN6 of the Ren meridian is the sea of qi, which has the effect of reinforcing yuan qi, tonifying kidney and consolidating essence. So, RN6 can help the acupoints of Yangming meridian to benefit qi. The Xuehai (SP10) is the acupoint of the spleen meridian of the foot Taiyin, which has the effect of invigorating blood circulation and unblocking blood flow. *A-B Classic of Acupuncture and Moxibustion* mentioned that “if the blood is obstructed…… Xuehai (SP10) will be taken.” The EX-LE10 and the EX-UE9 belongs to the extraordinary acupoints that have a strong effect of regulating local qi and blood and activating meridians and collaterals. In general, the principle of acupuncture treatment involves activating meridians and collaterals, benefiting qi, and promoting blood circulation to remove blood stasis.

A meta-analysis of 19 studies with 1,108 patients found that acupuncture for CIPN had a significant advantage in terms of overall efficacy (45) and that ST36, LI4, LI11, and LR3 were the most frequently used acupoints in terms of selection. Although there are many similarities in the selection of acupoints in these studies, there is no uniformity of criteria. The selection of acupoints for our study was guided by syndrome differentiation. According to this differentiation, the patients with a syndrome of qi deficiency and blood stasis were enrolled. The principle of acupuncture treatment involves activating meridians and collaterals, benefiting qi, and promoting blood circulation to remove blood stasis, which makes our treatment of CIPN patients more targeted and effective. Based on clinical findings in existence (27, 46–51), acupuncture is estimated to have potential therapeutic effect in treating symptoms of CIPN with particular advantages of relieving pain and improving the quality of life. Some studies are single-arm trials and deficient in randomization and control (49–51). Some studies used a blank control rather than a sham acupuncture group control (27). The results of this study also showed that acupuncture treatment effectively relieved pain and improved physical fitness for patients. To eliminate the potential placebo effect caused by the acupuncture itself, we created the sham acupuncture group. In addition, the verum acupuncture could alleviate TCM symptoms of patients with the syndrome of qi deficiency and blood stasis.

There are several important areas for improvement as follows. 1. The most significant limitation of the trial was the short intervention time, although our findings suggest certain effects after 2 weeks of treatment in the verum acupuncture group. We followed up the patients who had completed the treatment. Some patients chose to continue acupuncture treatment, and as the treatment time prolonged, the patients’ peripheral nerve-related symptoms were relieved more significantly. Based on this trial’s result and the overall positive trend, future studies may consider including longer treatment and follow-up phases. 2. This study was a small-sample single-center trial, and only patients from China were observed. We look forward to a larger multi-center trial to further verify our results.

## 5 Conclusion

This study supports the feasibility of acupuncture combined with medication as an intervention for patients with CIPN and determines its efficacy and safety in improving peripheral neuropathy. As there are no known effective treatments to prevent or reverse CIPN, the potential symptom management benefit of acupuncture in CIPN deserves further study in a large-sample multi-center trial with a long-term follow-up. Furthermore, exploring the predictive genetic risk markers associated with the development of CIPN may be crucial to improve the design of future clinical trials to test neuroprotective strategies targeting CIPN.

## Data Availability

The original contributions presented in the study are included in the article/supplementary material. Further inquiries can be directed to the corresponding author.
